# Correction: Associations of self-reported hearing problems with long-term trajectories of mental and functional health in middle-aged and older adults: The role of self-perceptions of aging

**DOI:** 10.1371/journal.pone.0335892

**Published:** 2025-10-31

**Authors:** Markus Wettstein, Ann-Kristin Reinhard, Bettina Williger, Susanne Wurm

The corresponding author, Markus Wettstein’s email address is incorrect. Markus Wettstein’s email address is: markus.wettstein@hu-berlin.de

There is an error in affiliation 1 for Markus Wettstein. The correct affiliation 1 is: Humboldt-Universität Zu Berlin, Germany.

The following information is missing from the Funding statement: We acknowledge support by the Open Access Publication Fund of Humboldt-Universität Zu Berlin.
