# Supplementary material for: Associations of self-reported hearing problems with long-term trajectories of mental and functional health in middle-aged and older adults: The role of self-perceptions of aging
Source: PLoS One. 2025 Sep 2;20(9):e0328814. doi: 10.1371/journal.pone.0328814 (PMC12404373; doi:10.1371/journal.pone.0328814)
Supplement: S1 File — (DOCX) [file pone.0328814.s001.docx]

Analytical Code (SAS)

Depressive Symptoms as Outcome:

**PROC** **MIXED** DATA=sen.deashoermultilevel (where = (depressiv ne **.** and fh ne **.** and alter_z ne **.** and contgrowth ne **.** and socloss ne **.** and physloss_c ne **.** and hoermean_c ne **.**

and hearaid ne **.** and sex_c ne **.** and anzphy_c ne **.** and bildung4_c ne **.** and westost ne **.**)) NOCLPRINT COVTEST MAXITER=**100** METHOD=ML;

CLASS fallnum;

model depressiv = alter_z sex_c hearaid anzphy_c

bildung4_c westost_c contgrowth_c socloss_c physloss_c hoermean_c hoerdev

contgrowth_c*alter_z

stichdummy1 stichdummy2 stichdummy3

contgrowth_c*hoermean_c socloss_c*hoermean_c physloss_c*hoermean_c contgrowth_c*hoerdev socloss_c*hoerdev physloss_c*hoerdev

time

alter_z*time sex_c*time

hoermean_c*time hearaid*time

anzphy_c*time

bildung4_c*time westost_c*time contgrowth_c*time socloss_c*time physloss_c*time

contgrowth_c*hoermean_c*time socloss_c*hoermean_c*time physloss_c*hoermean_c*time

stichdummy1*time stichdummy2*time stichdummy3*time

/SOLUTION DDFM=bw NOTEST CHISQ covb ;

RANDOM intercept time / SUBJECT=fallnum TYPE=UN;

store out = MixedModel;

**RUN**;

Functional Health as Outcome:

ods rtf;

**PROC** **MIXED** DATA=sen.deashoermultilevel (where = (depressiv ne **.** and fh ne **.** and alter_z ne **.** and contgrowth ne **.** and socloss ne **.** and physloss_c ne **.** and hoermean_c ne **.**

and hearaid ne **.** and sex_c ne **.** and anzphy_c ne **.** and bildung4_c ne **.** and westost ne **.**)) NOCLPRINT COVTEST MAXITER=**100** METHOD=ML;

CLASS fallnum;

model fh= alter_z sex_c hearaid anzphy_c

bildung4_c westost_c contgrowth_c socloss_c physloss_c hoermean_c hoerdev

contgrowth_c*alter_z physloss_c*alter_z

stichdummy1 stichdummy2 stichdummy3

contgrowth_c*hoermean_c socloss_c*hoermean_c physloss_c*hoermean_c contgrowth_c*hoerdev socloss_c*hoerdev physloss_c*hoerdev

contgrowth_c*hoermean_c*alter_z

time

alter_z*time sex_c*time

hoermean_c*time hearaid*time anzphy_c*time

bildung4_c*time westost_c*time contgrowth_c*time socloss_c*time physloss_c*time

socloss_c*time*alter_z

contgrowth_c*hoermean_c*time socloss_c*hoermean_c*time physloss_c*hoermean_c*time

socloss_c*hoermean_c*time*alter_z

stichdummy1*time stichdummy2*time stichdummy3*time

/SOLUTION DDFM=bw NOTEST CHISQ covb;

RANDOM intercept time / SUBJECT=fallnum TYPE=UN;

store out = MixedModel;

**RUN**;

ods rtf close;
