# Supplementary material for: Associations of self-reported hearing problems with long-term trajectories of mental and functional health in middle-aged and older adults: The role of self-perceptions of aging
Source: PLoS One. 2025 Sep 2;20(9):e0328814. doi: 10.1371/journal.pone.0328814 (PMC12404373; doi:10.1371/journal.pone.0328814)
Supplement: Attachment — Submitted filename: response letter.docx [file pone.0328814.s003.docx]

PONE-D-24-42847

Associations of Self-Reported Hearing Problems with Long-Term Trajectories of Mental and Functional Health in Middle-Aged and Older Adults: The Role of Self-Perceptions of Aging

PLOS ONE

October, 2024

Dear Soham Bandyopadhyay,

Thank you very much for your message. We are very grateful for the opportunity to revise the manuscript.

Notes regarding the specific revisions made in response to each comment are included below (indicated by “Response: …”). We hope that you will find the revised manuscript improved and responsive to the queries that had been made.

Sincerely yours,

On behalf of the authors

Markus Wettstein, PhD

Editor’s comments:

“Please provide written evidence from an ethical review board that ethics was not required for this survey of human participants before we can proceed to a formal review.“

Response: Thank you; we have added the following explaining text as on the submission website:

“Please not that this is a secondary data analysis. We used data from the German Ageing Survey, but we did not conduct this survey.

Ethical approval was not obtained because this is not mandatory for general surveys in

Germany. The German Ageing Survey does not use any invasive methods. The German Research Foundation (<https://www.dfg.de/de/foerderung/antrag-foerderprozess/faq/geistes-sozialwissenschaften#253024>) specifies criteria when ethical approval is mandatory in Germany (e.g., whenever patients are investigated or whenever the investigation comes with a physical risk for study participants). These criteria are not met in the German Ageing Survey, which is why ethical approval was not obtained. According to the Rat für Wirtschafts- und Sozialdaten (German Data Forum) „Empirical social and economic research in the German-speaking world has no tradition of having its projects reviewed by ethics committees” (<https://www.konsortswd.de/wp-content/uploads/RatSWD_Output9_Forschungsethik.pdf>; p. 28).

Rat für Wirtschafts- und Sozialdaten (2017). *Forschungsethische Grundsätze und Prüfverfahren in den Sozial-und Wirtschaftswissenschaften [Principles and Review Procedures of Research Ethics in the Social and Economic Sciences]* (No. 9 (5)). RatSWD Output.

The survey maintains an academic advisory board which ensures its scientific quality. Prior to

study participation, all study participants provided informed consent.

  
Comment: “When submitting your revision, we need you to address these additional requirements.

Please ensure that your manuscript meets PLOS ONE's style requirements, including those for file naming. The PLOS ONE style templates can be found at
https://journals.plos.org/plosone/s/file?id=wjVg/PLOSOne_formatting_sample_main_body.pdf and
https://journals.plos.org/plosone/s/file?id=ba62/PLOSOne_formatting_sample_title_authors_affiliations.pdf.”

Response: Thank you for raising this point; we believe that our file naming for the supporting information files was not correct, and we have changed that accordingly.

Comment**:** “Thank you for stating the following financial disclosure: "The German Ageing Survey (DEAS) was funded under Grant 301-6083-05/003*2 by the German Federal Ministry for Family, Senior Citizens, Women, and Youth."

Please state what role the funders took in the study. If the funders had no role, please state: "The funders had no role in study design, data collection and analysis, decision to publish, or preparation of the manuscript.

Please include this amended Role of Funder statement in your cover letter; we will change the online submission form on your behalf."

Response**:** We apologize if our statement was misleading, we changed it as follows:

“The author(s) received no specific funding for this work. The authors use data from the German Ageing Survey (DEAS, which was funded under Grant 301-6083-05/003*2 by the German Federal Ministry for Family, Senior Citizens, Women, and Youth.”

Comment**:** “Thank you for stating the following in the Acknowledgments Section of your manuscript: "The German Ageing Survey (DEAS) was funded under Grant 301-6083-05/003*2 by the German Federal Ministry for Family, Senior Citizens, Women, and Youth. The content is the sole responsibility of the authors."

We note that you have provided funding information that is not currently declared in your Funding Statement. However, funding information should not appear in the Acknowledgments section or other areas of your manuscript. We will only publish funding information present in the Funding Statement section of the online submission form.

Please remove any funding-related text from the manuscript and let us know how you would like to update your Funding Statement. Currently, your Funding Statement reads as follows: "The German Ageing Survey (DEAS) was funded under Grant 301-6083-05/003*2 by the German Federal Ministry for Family, Senior Citizens, Women, and Youth."

Please include your amended statements within your cover letter; we will change the online submission form on your behalf.”

Response: We would like to remove the Acknowledgments Section (please see revised manuscript), as the funding is already reported as part of the Funding Statement.

Comment**:** “We note that you have indicated that there are restrictions to data sharing for this study. For studies involving human research participant data or other sensitive data, we encourage authors to share de-identified or anonymized data. However, when data cannot be publicly shared for ethical reasons, we allow authors to make their data sets available upon request. For information on unacceptable data access restrictions, please see http://journals.plos.org/plosone/s/data-availability#loc-unacceptable-data-access-restrictions.

Before we proceed with your manuscript, please address the following prompts:

a) If there are ethical or legal restrictions on sharing a de-identified data set, please explain them in detail (e.g., data contain potentially identifying or sensitive patient information, data are owned by a third-party organization, etc.) and who has imposed them (e.g., a Research Ethics Committee or Institutional Review Board, etc.). Please also provide contact information for a data access committee, ethics committee, or other institutional body to which data requests may be sent.

b) If there are no restrictions, please upload the minimal anonymized data set necessary to replicate your study findings to a stable, public repository and provide us with the relevant URLs, DOIs, or accession numbers. Please see http://www.bmj.com/content/340/bmj.c181.long for guidelines on how to de-identify and prepare clinical data for publication. For a list of recommended repositories, please see https://journals.plos.org/plosone/s/recommended-repositories. You also have the option of uploading the data as Supporting Information files, but we would recommend depositing data directly to a data repository if possible.

Please update your Data Availability statement in the submission form accordingly.

Response**:** We have changed the Data Availability statement as follows:

“Given that this is a secondary data analysis, we are not in the position to share the data. The data from the German Ageing Survey can be obtained from the German Centre of Gerontology, Research Data Centre: <https://www.dza.de/en/research/fdz/access-to-data>". Our description of the measurement occasions that were used in the manuscript, data exclusions etc. will allow any researcher to build a dataset, based on the data received from the Research Data Centre of the German Centre of Gerontology, that will correspond to the dataset we used for analyses. We also provide the analytical code of our analyses to facilitate replication.

Comment**:** “5. Please include captions for your Supporting Information files at the end of your manuscript, and update any in-text citations to match accordingly. Please see our Supporting Information guidelines for more information: <http://journals.plos.org/plosone/s/supporting-information>.”

Response**:** We have added captions for our Supporting Information file at the end of the manuscript.

Comment: “While revising your submission, please upload your figure files to the Preflight Analysis and Conversion Engine (PACE) digital diagnostic tool, [https://pacev2.apexcovantage.com/](https://c05y1x9s.r.us-east-2.awstrack.me/L0/https:%2F%2Fpacev2.apexcovantage.com%2F/1/010f01929b4d7894-dc285029-6a39-49ab-85d3-931ac636c1f1-000000/lSTQIoVrqXyo8aDIGx_8Pqym5QE=181). PACE helps ensure that figures meet PLOS requirements. To use PACE, you must first register as a user. Registration is free. Then, login and navigate to the UPLOAD tab, where you will find detailed instructions on how to use the tool. If you encounter any issues or have any questions when using PACE, please email PLOS at [figures@plos.org](mailto:figures@plos.org). Please note that Supporting Information files do not need this step.”

Response: We have uploaded the figures on the PACE website, and there were no comments, so we believe that the figures meet PLOS requirements.
